# Ozone Exposure and Asthma Attack in Children

**DOI:** 10.3389/fped.2022.830897

**Published:** 2022-04-05

**Authors:** Wanting Huang, Jinzhun Wu, Xiaoliang Lin

**Affiliations:** ^1^Women and Children’s Hospital, School of Medicine, Xiamen University, Xiamen, China; ^2^The First Affiliated Hospital of Xiamen University, Xiamen, China

**Keywords:** asthma attack, ozone, child, case-crossover design, air pollution

## Abstract

**Background:**

Increasing evidence indicated that ozone (O_3_) exposure could trigger asthma attacks in children. However, the effect of O_3_ at low concentrations is uncertain.

**Purpose:**

This study aimed to explore the effects of O_3_ exposure at low concentrations on asthma attacks in children.

**Methods:**

A total of 3,475 children with asthma attacks from the First Affiliated Hospital of Xiamen University were available for the analyses. Air pollution data and meteorological data in Xiamen during 2016–2019 were also collected. A case-crossover design and conditional logistic regression models were conducted to evaluate the association between asthma attacks and outdoor air pollution with lag structures (from lag 0 to lag 6) in both single and multi-pollutant models. Furthermore, we estimated the influence of various levels of O_3_ exposure on an asthma attack in three groups categorized by maximum daily 8-h sliding average ozone (O_3_-8 h) (O_3_-8 h ≥ 100 μg/m^3^, O_3_-8 h: 80–99 μg/m^3^, O_3_-8 h < 80 μg/m^3^).

**Results:**

For both single-pollutant models and multi-pollutant models, when O_3_-8 h was higher than 80 μg/m^3^, O_3_ exposure was increased the risk of acute asthma attacks on each day of lag. The effect of O_3_ on children with asthma was significant when O_3_ concentration was higher than 100 μg/m^3^.

**Conclusion:**

O_3_ concentration above 80 μg/m^3^ contributed to an increased risk of asthma attacks in children.

## Introduction

Bronchial asthma is a common chronic disease in childhood with shortness of breath, wheezing, and coughing from constriction and mucous-membrane swelling in the bronchi (1). It is estimated that the prevalence of asthma among children aged 0–14 years in China in 2010 was 3.02% (95% confidence interval (CI): 2.97–3.06%) (2). Although most children with asthma can be well-controlled through standardized treatment, many children still need rescue medication or hospitalization for acute asthma attacks. Avoiding the risk factors and predisposing factors is essential for the effective management of asthma. The risk factors for acute asthma attacks are complex, including dust mites, fungi, pollen, and infectious factors. In recent years, air pollutants have gradually attracted attention. Ozone (O_3_), one of the air pollutants, has been repeatedly reported to be associated with respiratory diseases such as asthma (3) when the ozone concentration is too high.

O_3_ exists in the stratosphere and troposphere. When in the stratosphere, it prevents ultraviolet rays from harming the human body. However, when in the troposphere, it adversely affects the human respiratory and cardiovascular system when it reaches high concentrations. Ozone is a strong oxidizing gas, easily soluble in water, and the inhaled ozone can penetrate deeply into the lungs (4). Some studies found that ozone might cause airway inflammation, worse lung function, airway hyperresponsiveness, and increased sensitivity to allergens (5–7). According to National Ambient Air Quality Standard for Ozone, the daily maximum 8-h average ozone concentration should be less than 150 μg/m^3^, and the World Health Organization believes that the daily maximum 8-h average ozone concentration should be less than 100 μg/m^3^.

Previous studies have suggested that O_3_ exposure only affect children with asthma (including increasing respiratory symptoms, asthma medication use, and impaired lung function) in the most polluted areas (8). Some recent studies have shown that even if it is lower than the standard set by the World Health Organization, O_3_ exposure may also cause an acute attack of asthma in children. In a Quebec birth cohort study, the average ozone concentration during the study period was 32.07 ppb (68.76 μg/m^3^). This study found that ozone exposure was associated with the risk of asthma, with an odds ratio (OR) value of 1.11 (95% CI: 1.10, 1.12) (9). Short-term increases in low levels (<70 ppb) of ozone are associated with decreased lung function (10). Children with asthma who use maintenance medications are particularly susceptible to the effects of ozone below the US Environmental Protection Agency (EPA) standard (11).

Xiamen is a city on the southeast coast of China. During 2016–2019, the average concentration of maximum daily 8-h sliding average ozone (O_3_-8 h) was 81.21 μg/m^3^, the proportion of ozone concentration above ozone level 2 standard (160 μg/m^3^)was 0.62%. In the present study, the effects of increasing ozone levels at low concentrations on acute asthma attacks were investigated.

## Methods

### Study Participants

There were 29 065 cases with the diagnosis code J45 in the emergency department of the First Affiliated Hospital of Xiamen University from 1 January 2016 to 31 December 2019. According to the “Guidelines for the Diagnosis and Treatment of Children’s Bronchial Asthma (2016 Edition),” a total of 3,959 cases were identified as acute asthma attacks. After excluding 221 (5.58%) cases with fever, 15 (0.38%) with acute asthma attacks caused by self-discontinuation within 14 days, 7 cases with allergen exposure, and 2 cases (0.05%) outside of the region, a total of 3,714 cases (93.81%) of acute asthma attacks were eligible for the analyses.

### Medical Data

Data were obtained from the Electronic Medical Record for asthma (code J45, International Classification of Diseases, Tenth Revision—ICD-10) among children aged 0-14 years in the First Affiliated Hospital of Xiamen University from 1 January 2016 to 31 December 2019. Combined with the main complaint, current medical history, and time of visit, the date of the first acute onset of asthma in the child was determined, and the recurrent cases were counted as one. The data of non-local residents and cases with fever and a clear diagnosis of asthmatic bronchitis were excluded.

### Air Pollution

Ambient monitoring data, including O_3_-8 h concentrations and 24-h average concentrations of carbon monoxide (CO), nitrogen dioxide (NO_2_), sulfur dioxide (SO_2_), and particulate matter (PM_10_ and PM_2.5_), were measured at four monitoring stations in Xiamen metropolitan from 1 January 2016 to 31 December 2019. The 8-h average concentration refers to the average concentrations of continuous 8 h, and the daily maximum 8-h average concentration of zone refers to the maximum value of the continuous 8-h average. For example, N1 = average {c1, c2, c3, c8}, N2 = average {c2, c2, c3, c9}, N3 = average {c3, c2, c3, c10}, and so on, N17 = average {c17, c2, c3, c24}. The daily maximum 8-h average concentration = max {N1, N2, N3, N17}. The pollutants concentration was measured by the same method at each monitoring station. O_3_, NO_2_, and SO_2_ concentrations were measured by using an open-path deferential optical absorption spectroscopy (DOAS) instrument.

### Meteorological Data

Meteorological data, including daily average temperature (in degrees centigrade), relative humidity (as a percentage), wind velocity (in meters per second), and rainfall (in millimeter) were collected from the Xiamen Meteorological Bureau.

### Statistical Methods

In this study, the case-crossover method was used to explore the relationship between ozone and asthma exacerbations. Case-crossover design has been widely used in environmental epidemiology to study the impact of short-term environmental exposure on the risk of individuals with rapid onset events. Since each case served as their control and matches all time-varying, unmeasured or unmeasured thematic characteristics, a case-crossover design eliminates time-constant and slowly varying confounders (such as gender, race, age, genetics, personal lifestyle, or behavior). This study was a retrospective analysis and the approaches was “symmetric two-way.” The date of the asthma attack was the case date, and two control dates were the date 1 week before the case date and the other after, avoiding the day of the week effect.

Considering the lag effect of the concentrations of each pollutant, lag0 to lag6 was selected. To control the potential confounding effect of weather, we set the daily relative humidity, average temperature, average wind speed, and rainfall in the same period lag 0–6 days as covariates. Considering the possible interaction between ozone and other pollutants, we also discussed the relationship between acute asthma attacks and other gaseous pollutants (NO_2_, CO, and SO_2_) and particulate pollutants (PM_10_ and PM_2.5_).

The analysis strategy of each part of the correlation model is as follows:

(1) Single-pollutant model: After adjusting for the meteorological factors, single-pollutant models of O_3_ from lag 0 to lag 6 were established.
(2) Multi-pollutant model: After controlling for confounding factors, a multi-pollutant model of O_3_ + NO_2_ + CO + SO_2_ + PM_2.5_ + PM_10_ was established and the OR with a 95% CI was calculated.
(3) Ozone concentration grouping model: lag structures was categorized into three groups according to the O_3_-8 h concentrations (O_3_-8 h ≥ 100 μg/m^3^, O_3_-8 h: 80–99 μg/m^3^, O_3_-8 h < 80 μg/m^3^). After adjusting for the lagging meteorological factors, the single-pollutant and multi-pollutant model were established and the effects of pollutants on acute asthma attacks were calculated.

Conditional logistic regression models were used to identify associations between air pollutants and asthma attacks in children, and all results were expressed as OR and 95% CI. All statistical analyses were performed in the R (version 3.6.0).

## Results

### Characteristics of Acute Asthma Attacks and Participants

The characteristics of acute asthma attacks are presented in Table 1. Male children accounted for 69.15% of cases, and cases younger than 6 years old accounted for 60.07%. The diagnosis included eight types of J45. Among them, a total of 18 children were hospitalized, and the rest were treated in outpatient clinics and followed up. There were no deaths.

**TABLE 1:** Characteristics of acute asthma attacks from 2016 to 2019.

	Acute asthma attacks (*N* = 3,714)	Percentage (%)
**Gender**		
Female	1,146	30.85
Male	2,569	69.15
**Age**		
<6 years old	2,231	60.07
≥6 years old	1,483	39.93
**Diagnosis (ICD-10)**		
J45.000, allergic asthma	2	0.05
J45.003, allergic bronchial asthma	1	0.03
J45.004, allergic rhinitis with asthma	11	0.30
J45.005, cough variant asthma	448	12.06
J45.006, childhood asthma	664	17.88
J45.900, asthma	277	7.46
J45.903, bronchial asthma, non-critical	2,304	62.04
J45.904, bronchial asthma, critically ill	7	0.19
**Visiting status**		
First visit	2,281	61.42
Follow-up	1,433	38.58
**Treatment**		
Hospitalization	18	0.48
Stay in the hospital for observation	6	0.16
Outpatient treatment and follow-up	3,690	99.35

Among the 3,714 cases, there were 3,475 patients, including 2,383 males (68.58%) and 1,092 females (31.42%). Children younger than 6 years old accounted for 61.41%. There were 779 (22.42%) children with a history of eczema, 360 (10.36%) children with rhinitis. There were 784 (22.56%) patients with rhinitis in the family, and 110 (3.17%) patients with a history of asthma, as shown in Table 2.

**TABLE 2:** Characteristics of the study participants.

	Number of patients (*N* = 3,475)	Percentage (%)
**Gender**		
Female	1,092	31.42
Male	2,383	68.58
**Age**		
<6 years old	2,134	61.41
≥6 years old	1,341	38.59
**Past history**		
Rhinitis	360	10.36
Eczema	779	22.42
**Family history**		
Rhinitis	784	22.56
Asthma	110	3.17
Urticaria	35	1.00

### Characteristics of Air Pollutant Concentrations and Meteorological Variables

As shown in Table 3, the average concentration of O_3_-8 h in Xiamen during 2016–2019 was 81.21 μg/m^3^, the proportion of ozone concentration above ozone second-level standard (160 μg/m^3^) was 0.62% and the number of days exceeding ozone first-level standard (100 μg/m^3^) was 361 days (24.7%). The average concentrations of NO_2_, PM_2.5_, and PM_10_, were 29.02, 12.057, and 44.85 μg/m^3^, respectively, and the proportion of days exceeding the first-level standard was 0.34, 7.94, and 33.26%, respectively. The daily average concentrations of CO and SO_2_ did not exceed the first-level standard.

**TABLE 3:** Characteristics of daily air pollutants and meteorological variables in Xiamen from 2016 to 2019.

	Mini	Max	Average	Q1	Q2	Q3	IQR
O_3_-8 h (μg/m^3^)	12	178	81	60	79	99	39
CO (mg/m^3^)	0.2	1.6	0.6	0.5	0.6	0.7	0.2
SO_2_ (μg/m^3^)	2	35	9	6	8	11	5
NO_2_ (μg/m^3^)	6	121	29	19	26	36	17
PM_2.5_ (μg/m^3^)	0.006	97.000	12.057	0.024	0.110	22.000	21.976
PM_10_ (μg/m^3^)	10	141	44	30	41	56	26
Temp (^°^C)	3.9	32.2	21.6	16.5	22.1	27.2	10.7
Rain (mm)	0	172.7	4.1	0	0	0.9	0.9
Wind (m/s)	0.6	9.6	2.7	2.0	2.5	3.2	1.2
Humi (%)	27	99	77.43	69	79	87	18

*IQR, Interquartile range.*

*IQR = Q3 - Q1.*

### Correlation Analysis of Air Pollutant Concentrations and Meteorological Variables

As shown in Table 4, there was a positive correlation between ozone and SO_2_, PM_2.5_, PM_10_, air temperature, and wind speed (*P* < 0.05), and the correlation coefficients were 0.125, 0.418, 0.418, 0.124, and 0.118, respectively. There was a negative correlation between relative humidity, rainfall, and ozone (*P* < 0.05).

**TABLE 4:** Correlation analysis between air pollutants and meteorological factors in Xiamen from 2016 to 2019.

	O_3_-8 h	CO	SO_2_	NO_2_	PM_2.5_	PM_10_	Humi	Temp	Rain	Wind
O_3_-8 h	1.000									
CO	0.029	1.000								
SO_2_	0.125*	0.356*	1.000							
NO_2_	−0.042	0.597*	0.618*	1.000						
PM_2.5_	0.418*	0.264*	0.094*	0.138*	1.000					
PM_10_	0.418*	0.497*	0.609*	0.503*	0.367*	1.000				
Humi	−0.492*	0.108*	−0.067*	0.174*	−0.166*	−0.408*	1.000			
Temp	0.124*	−0.442*	0.032	−0.300*	−0.206*	−0.277*	0.133*	1.000		
Rain	−0.358*	0.141*	−0.244*	0.116*	−0.173*	−0.387*	0.646*	−0.106*	1.000	
Wind	0.118*	−0.313*	0.138*	−0.501*	−0.248*	−0.226*	−0.375*	−0.077*	−0.069*	1.000

*P < 0.05.

### Single Pollutant Model

**FIGURE 1:**
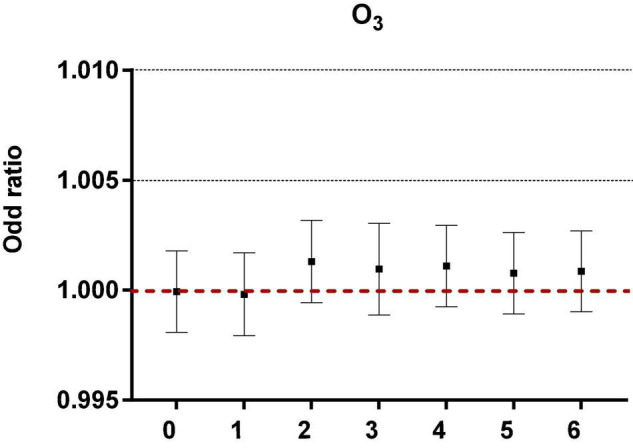
The correlation of ozone in different lag periods to acute asthma attacks in the single pollutant model.

After controlling the confounding factors of daily relative humidity, average temperature, average wind speed, and rainfall, the single pollutant models were established, as shown in Figure 1.

### Multi-Pollutant Model

**FIGURE 2:**
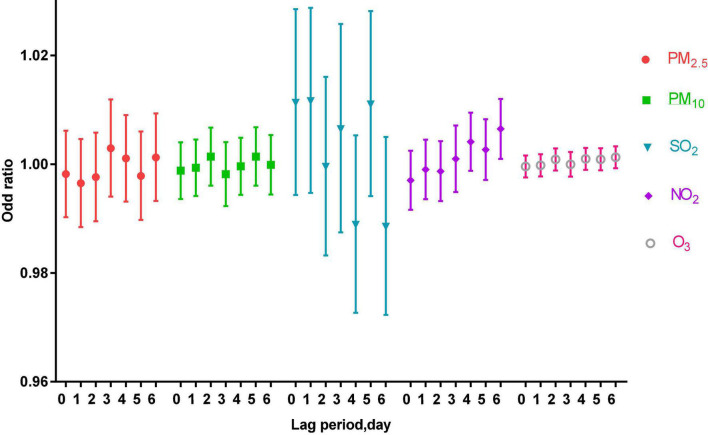
The correlation of air pollutants in different lag periods to acute asthma attacks in the multi-pollutant model.

Under the adjustment of meteorological factors and weekly effects, a multi-pollutant model was constructed to evaluate the effects of pollutants on acute asthma attacks, as shown in Figure 2.

### Analysis of Ozone Concentration in Different Lag Periods

#### Single Pollutant Model

**FIGURE 3:**
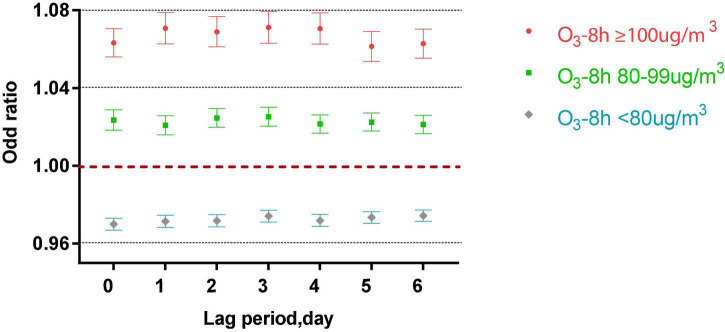
Different O_3_-8 h levels in different lag periods in the single pollutant model.

Figure 3 shows that in the single-pollutant model that adjusts for the lagging meteorological factors, acute asthma attacks were associated with different lag periods and different levels of ozone exposure (*P* < 0.001). When O_3_-8 h is greater than or equal to 100 μg/m^3^, every 10 μg/m^3^ increase of O_3_-8 h increased 6.33, 7.08, 6.89, 7.11, 7.06, 6.14, and 6.28% of acute asthma attacks from 0 to 6 days lag.

When O_3_-8 h was between 80 and 99 μg/m^3^, every 10 μg/m^3^ increase of O_3_-8 h increased 2.36, 3.08, 2.46, 2.52, 2.15, 2.25, and 2.13% of acute asthma attacks from 0 to 6 days lag.

From lag 0 to lag 6, when O_3_-8 h is less than 80 μg/m^3^, every 10 μg/m^3^ increase in O_3_-8 h, the odds ratios is 0.9700, 0.9714, 0.9717, 0.9741, 0.9719, 0.9734, and 0.9743.

#### Multi-Pollutant Model

**FIGURE 4:**
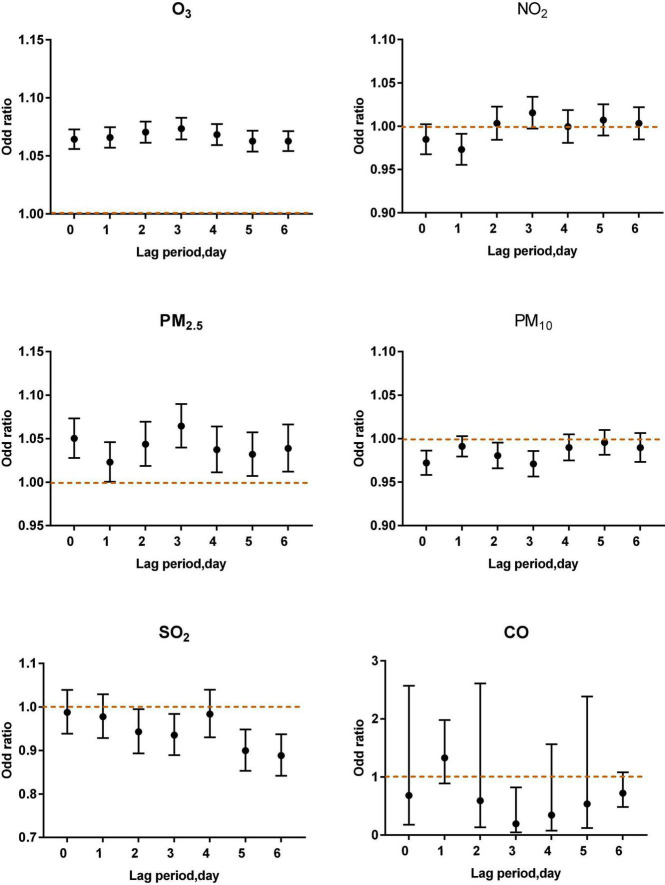
Air pollutants in different lag periods in the multi-pollutant model when O_3_-8 h was higher than 100 μg/m^3^.

**FIGURE 5:**
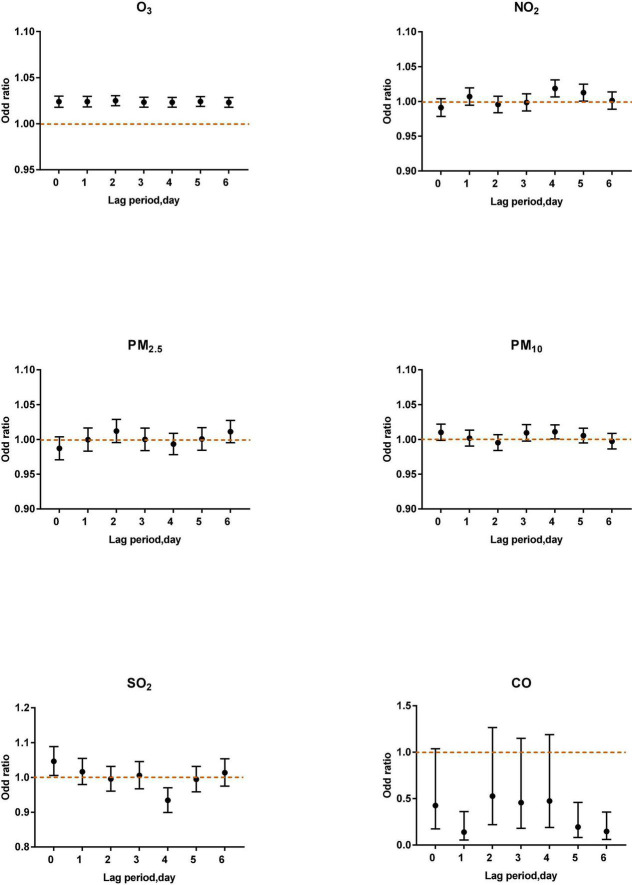
Air pollutants in different lag periods in the multi-pollutant model when O_3_-8 h was between 80 and 99 μg/m^3^.

**FIGURE 6:**
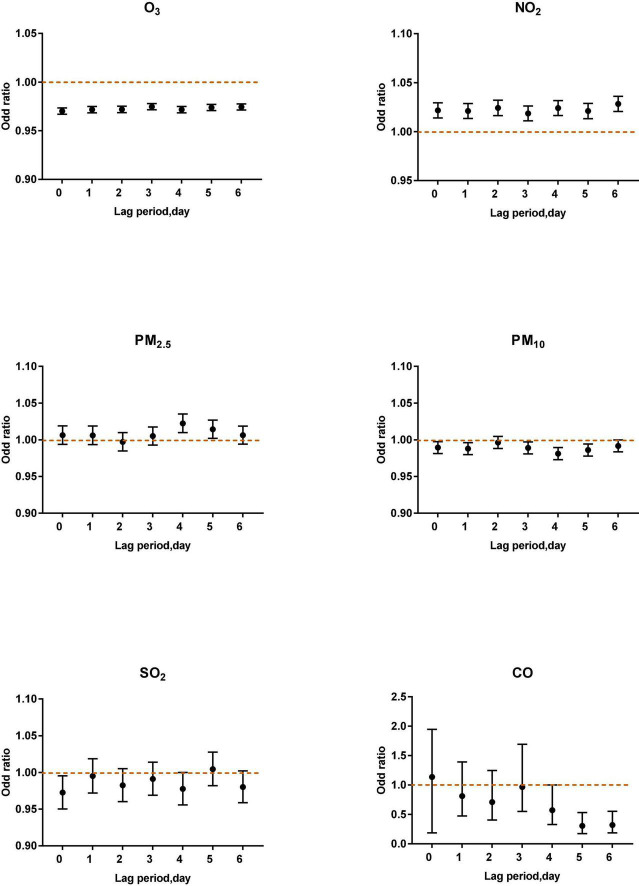
Air pollutants in different lag periods in the multi-pollutant model when O_3_-8 h was less than 80 μg/m^3^.

As shown in Figures 4–6, the multi-pollutant model with different ozone levels in different lag periods showed that the health effects of ozone on asthma attacks in children was similar to that in the single-pollutant model. Ozone concentration above 80 μg/m^3^ contributed to an increased risk of asthma attacks in children. The effect of ozone on children with asthma was significant when ozone concentration was higher than 100 μg/m^3^. When ozone concentration was less than 80 μg/m^3^, the ozone concentration was negatively correlated with asthma attacks in children.

When O_3_-8 h was higher than 100 μg/m^3^, PM_2.5_ (lag0, OR: 1.0503, 95% CI: 1.0277–1.0733; lag2, OR: 1.0437, 95% CI:1.0186–1.0694; lag3 OR: 1.0644, 95% CI: 1.0398–1.0897; lag4, OR: 1.0372, 95% CI: 1.0112–1.0639; lag5, OR: 1.0319, 95% CI: 1.0071–1.0573; lag6, OR: 1.0388, 95% CI: 1.0121–1.0663) was positively correlated with asthma attacks. PM_10_ (lag0, lag2, lag3), SO_2_ (lag3, lag5, lag6), NO_2_ (lag1) were negatively associated with acute asthma attacks (Figure 4).

When O_3_-8 h was between 80 and 99 μg/m^3^, NO_2_ (lag4, OR: 1.0186, 95% CI: 1.0065–1.0309) was positively correlated with asthma attacks. SO_2_ (lag4, OR: 0.9341, 95% CI: 0.8993–0.9703) was negatively associated with acute asthma attacks (Figure 5).

When O_3_–8 h was less than 80 μg/m^3^, PM_2.5_ (lag4, OR: 1.0223, 95% CI: 1.0097–1.0351) and NO_2_ (lag0, OR: 1.0216, 95% CI: 1.0140–1.0294; lag1, OR: 1.0210, 95% CI: 1.0134–1.0287; lag2, OR: 1.0242, 95% CI: 1.0164–1.0321; lag3 OR: 1.0186, 95% CI: 1.0111–1.0261; lag4, OR: 1.0241, 95% CI: 1.0165–1.0317; lag5, OR: 1.0210, 95% CI: 1.0132–1.0289; lag6, OR: 1.0283, 95% CI: 1.0206–1.0361) were positively correlated with asthma attacks. PM_10_ (lag0, lag1, lag2, lag3), SO_2_ (lag0) were negatively associated with acute asthma attacks (Figure 6).

The above correlations were all statistically significant.

## Discussion

### Ozone and Asthma

High levels of ozone are a risk factor for childhood asthma attacks. Pulmonary function tests were used to evaluate the prevalence of asthma in children in areas with high and low ozone concentrations, and it was found that ozone pollution would increase the prevalence of asthma (12). Moreover, high levels of ozone may increase the number of hospitalizations in children with asthma (13). A study from South Texas that included 902 children who were hospitalized for asthma at least twice found that a higher level of ozone was significantly associated with an increased number of children hospitalized for asthma. A foreign birth cohort study found that long-term exposure to high levels of ozone (>70 ppb) was also associated with hospitalization in children with asthma (OR 1.16–1.68) (14). In addition, previous epidemiological studies have also reported that children with asthma who take maintenance drugs are particularly susceptible to high concentrations of ozone. The background levels of ozone in this study are O_3_-1 h average 59 ppb (SD: 19 ppb) and O_3_-8 h average 51 ppb (SD: 19 ppb). The study found that in the model of PM_2.5_ + O_3_, every 50 ppb increase in O_3_-1 h might increase the risk of wheezing and chest tightness by 35 and 47%, respectively (11).

Exposure to the high levels of ozone might lead to innate lymphoid cells (ILC2)-mediated type 2 immunity in children with asthma (15). T and B cells play a role in the adaptive immune. However, an animal study found that the upper respiratory tract changes induced by repeated exposure to ozone were mediated by the second group of ILC2, not T cells and B cells. In addition, surfactant protein D (SP-D) is a product of airway epithelial cells and plays an important role in immune defense mechanisms. SP-D can resist inflammatory changes and has a multimeric structure that is easily oxidized. Inhaled corticosteroids, which could induce the synthesis of SP-D have anti-inflammatory effects on the lungs and can effectively treat asthma (16). However, an animal study showed that ozone exposure weakened the anti-inflammatory effect of budesonide. Ozone exposure inhibited the release of SP-D in the airway.

As shown in Figures 3, 5, this study found that relatively low-level ozone (O_3_-8 h: 80–99 μg/m^3^) was harmful to acute asthma attacks. Studies have explored the effects of ozone exposure close to or lower than the US EPA standard on infant respiratory system. Previously study found that ozone exposure increased the risk of wheezing and dyspnea in infants whose mothers were diagnosed with asthma. The risk of wheezing and dyspnea increased by 59% (95% CI, 1–154%) and 83% (95% CI, 42–136%) for each additional Inter-quartile range (IQR) respectively (17). In recent years, some studies have also found the effects of a relatively low ozone environment on children with asthma. A case-crossover study collected the number of emergency department visits (*n* = 91 386) for asthma or wheezing in 41 hospitals for children aged 5–17 years. The Poisson generalized linear model found that even at relatively low ozone concentrations (47.3 ppb), ozone was still associated with childhood asthma or wheezing (18). In a cohort study focused on asthma drug treatment, an electronic drug monitor was installed on a short-acting Beta2 agonist (SABA) metered-dose inhaler to monitor the use of SABA. This study showed that ozone exposure below the US EPA standard was positively correlated with SABA use in both children and adults. Each interquartile range increase in ozone (16.8 ppb) increased the use of SABA in children (11.3%; 95% CI: 7.0–18.2%) than that in adults (8.4%; 95% CI: 6.4–11.0%) (19). Besides, the low ozone concentrations in the urban area might increase the responsiveness to allergens in patients with atopic asthma (20).

For both the single-pollutant model and multi-pollutant model (Figures 3, 6), when ozone concentration was less than 80 μg/m^3^, the ozone concentration was negatively correlated with asthma attacks in children. The potential mechanism of the protective effect of ozone at lower concentrations is still unclear. It might be related to the reactive oxygen species (ROS), produced by ozone, which has a dual influence on cell integrity. ROS are beneficial to cells at low concentrations (21). After ozone exposure, damage to the bronchiolar cell and airway inflammatory reaction is reversible. The study explored the influence of the interaction between genes and environmental factors on children with asthma observed that children with asthma who carry the TNF-308 GG gene have a significantly lower risk of bronchial symptoms under low ozone exposure (OR: 0.53, 95% CI: 0.31–0.91) (22). In addition, studies have found that oxidant genes have a protective effect on children living in low-ozone communities. A cohort study found that the effect of HMOX-1 gene variants on the risk of new-onset asthma varies with environmental ozone levels. The shorter HMOX-1 alleles (less than 23 repetitions) have the protective effect (HR, 0.44; 95% CI, 0.23–0.83) in children living in a low-level ozone environment (23).

In addition, indoor pollution has gradually entered the field of vision, and no association has been found between asthma and indoor ozone. A recent study from Beijing, China evaluated children’s cardiopulmonary response to indoor ozone exposure. During the study period, the average indoor ozone concentration (SD) was 8.7 (6.6) ppb, which was lower than the current guidelines and standards. The results showed low levels of indoor ozone were associated with decreased cardiac autonomic nerve function and increased heart rate in children. For every increase in IQR, cardiac autonomic nerve function decreases by −7.8% (95% CI: −9.9%, −5.6%), and heart rate increases by 2.6% (95% CI: 1.6%, 3.6%), but no significant correlation was found between the effects of airway inflammation and lung function (24).

### Other Pollutants and Asthma

Reports show that air pollution is a risk factor leading to global morbidity and mortality. Pollutants can induce a variety of respiratory symptoms and are closely related to respiratory diseases. Many studies have shown that it was significantly associated with asthma outcomes, such as morbidity, prevalence, hospitalization rate, emergency room visits, mortality, and asthma attacks (25). This study explores the effects of both gaseous pollutants and particulate pollutants on acute asthma attacks. The results showed the association between acute asthma attacks and PM_2.5_, PM_10_, NO_2_, and SO_2_ in the single-pollutant and multi-pollutant models. The results of a meta-analysis which included 22 case-crossover studies showed that all pollutants except SO_2_ and PM_10_ were significantly related to asthma attacks. The OR of ozone was 1.032 (95% CI: 1.005, 1.060) (26). A domestic study investigated 4,454 deaths from asthma in China from 2013 to 2018 by using case cross-analysis and conditional logistic regression model. The results showed that PM_2.5_ (lag 3; IQR, 47.1 μg/m^3^), NO_2_ (lag 3; IQR, 26.3 μg/m^3^) and O_3_ (lag 3; IQR, 52.9 μg/m^3^) were positively correlated with asthma mortality, with OR of 1.07 (95% CI): 1.01–1.22), 1.11 (95% CI: 1.01–1.22), and 1.09 (95% CI: 1.01–1.18), indicating that short-term exposure to pollutants may increase asthma mortality (27). Even exposure early in life may be related to the development of childhood asthma. A foreign prospective birth cohort study, which included 14 126 participants and followed up for 14–16 years found that the increase in PM_2.5_ and NO_2_ was associated with an increase in the risk of asthma. For every 10 μg/m^3^ increase, the OR values were 1.13 (95% CI 1.02–1.25) and 1.29 (1.00–1.66), respectively (28).

The effects of particulate pollutants on acute asthma attacks are consistent with findings from many previous studies (29, 30). We are facing the challenge of the combined effects of the environment and health. In this study, the daily average concentration of PM_2.5_ between 2016 and 2019 was 12.057 μg/m^3^, of which 116 days exceeded the first-level standard 35 μg/m^3^ (accounting for 7.94%). The average PM_10_ was 44.85 μg/m^3^, and the number of days exceeding the standard accounted for 33.26%. The average value of O_3_-8 h was 81.21 μg/m^3^, and the number of days exceeding the standard accounted for 24.7%. Controlling the balance between PM_2.5_ and ozone, and reducing the impact of particulate pollutants and ozone on health was still needed to be illustrated in the future. Merely reducing PM will also increase ozone, especially in winter. Merely reducing PM will also increase ozone, especially in winter. By reducing particulate pollutants, reducing the aerosol optical depth by 50% can increase ozone by 25% and also enhance the effect of volatile organic compounds (VOC) (31).

A study in North China using the Goddard Earth Observation System chemical transport model showed that PM_2.5_ might stimulate the production of ozone by slowing down the aerosol deposition of hydroperoxide radicals (8). The impact of short-term exposure to particulate pollutants and ozone on acute asthma attacks might vary depending on the asthma phenotype, such as whether it was associated with allergic diseases. A time-stratified case-crossover study found that in the two pollutant models with and without allergic diseases, when the 3-day moving average of ozone (lag 0–2) increased by 20 ppb, the OR was 1.08 (95%CI: 1.02, 1.14) and 1.00 (95% CI: 0.95, 1.05), for every 10 μg/m^3^ increase in PM_2.5_ 3-day moving average (lag 0–2), the odds ratio was 1.10 (95% CI: 1.07, 1.13) and 1.05 (95% CI: 1.02, 1.09), suggesting that asthma patients with allergic diseases were more susceptible to PM_2.5_ and ozone (32). In addition, exposure to particulate pollutants may be associated with inflammation of the lower respiratory tract. An animal experimental study found that PM_2.5_ (lag by 2 and 3 weeks) per IQR increased the risk of granulocytosis in bronchoalveolar lavage fluid by 11% (*p* = 0.04, 95% CI = 1.01–1.22) (33).

NO_2_ also affects children with asthma, such as asthma symptoms, decreased response to bronchodilators, and lung function damage. Studies have shown that asthma has the strongest association with O_3_ and NO_2_ (34). A systematic review of short-term exposure to O_3_ and NO_2_ found that daily short-term exposure to O_3_ and NO_2_ increased the risk of asthma exacerbation (35). We found that when O_3_-8 h was between 80 and 99 μg/m^3^, NO_2_ (lag 4 days) was positively correlated with the risk of an acute asthma attack. When O_3_-8 h was less than 80 μg/m^3^, NO_2_ had a significant positive correlation with the risk of an acute asthma attack after 0–6 days of lag, while ozone shows a negative correlation. Environmental ozone is mainly formed through complex photochemical reactions, and its precursors include nitrogen oxides (NOX: NO and NO_2_) and volatile organic compounds. The ozone level has a complex non-linear relationship with the concentration of NO_2_, and NO_2_ will weaken the photochemical reaction of O_3_ to a certain extent. When the NOX concentration is low, the ozone concentration increases with the increase in the NOX concentration and has nothing to do with the VOC concentration. When the NOX concentration is high, reducing NOX will increase ozone (31, 36). A study has shown that NO_2_ and O_3_ are closely related, and there is a long-term time sequence in some periods (37).

Sulfur dioxide can cause bronchoconstriction (5). A study in China pointed out that sulfur dioxide is related to the prevalence and symptoms of asthma in children (especially children with atopic allergies) (38) when the concentration of sulfur dioxide exceeded the World Health Organization’s Clean Air Guidelines. The results of this study showed thatSO_2_ (lag 0) was positively correlated with an acute asthma attack (OR: 1.0461, 95% CI: 1.0055–1.0884) when O_3_-8 h was between 80 and 99 μg/m^3^. Ozone is an effective oxidant, while sulfur dioxide is a reducing agent. They may cause asthma symptoms through different mechanisms.

### Advantages and Limitations

There are some advantages to this study. First of all, this study analyzed the influence of increasing ozone levels at low concentrations on acute asthma attacks. Secondly, the case-crossover study design is suitable for assessing the transient effects on the risk of the onset of acute events. Moreover, this design allows each patient to serve as his control. Patient-level confounding factors and time-varying variables are readily controlled. Finally, this study used single and multiple pollutant models to examine the relationship between ozone and asthma exacerbations.

The current research has some potential limitations. First, ambient concentration data were from only four monitoring stations of Xiamen. Ambient air monitoring equipment measures air quality at fixed outdoor locations, while asthma children breathe air in several indoor and outdoor environs throughout the day—ultimately do not fully represent children’s exposures. Second, this study did not include pollen exposure in the study. Pollen exposure in the daily environment may also affect lung function. Finally, this study is a single-center study. A multi-center study is needed to verify the reliability of the results.

## Conclusion

Our data provided a unique opportunity to examine the influence of ambient ozone on asthma attacks in children. The data indicate that short-term exposure to O_3_, PM_2.5_, PM_10_, NO_2_, and SO_2_ may be associated with acute asthma exacerbations. When ozone concentrations are higher than 80 μg/m^3^, children are at significantly increased risk of asthma attacks.

## Data Availability Statement

The original contributions presented in the study are included in the article/supplementary material, further inquiries can be directed to the corresponding author.

## Ethics Statement

The studies involving human participants were reviewed and approved by the Ethical Review Board of The First Affiliated Hospital of Xiamen University. Written informed consent from the participants’ legal guardian/next of kin was not required to participate in this study in accordance with the national legislation and the institutional requirements.

## Author Contributions

WH and JW coordinated and supervised the data. WH wrote the manuscript. JW and XL revised the manuscript. All authors contributed to the conception, interpretation of the results, and approved the final manuscript.

## Conflict of Interest

The authors declare that the research was conducted in the absence of any commercial or financial relationships that could be construed as a potential conflict of interest.

## Publisher’s Note

All claims expressed in this article are solely those of the authors and do not necessarily represent those of their affiliated organizations, or those of the publisher, the editors and the reviewers. Any product that may be evaluated in this article, or claim that may be made by its manufacturer, is not guaranteed or endorsed by the publisher.
